# Novel Two-Step Process in Cellulose Depolymerization: Hematite-Mediated Photocatalysis by Lytic Polysaccharide Monooxygenase and Fenton Reaction

**DOI:** 10.1021/acs.jafc.2c02445

**Published:** 2022-08-03

**Authors:** Damao Wang, Mu-Rong Kao, Jing Li, Peicheng Sun, Qijun Meng, Anisha Vyas, Pi-Hui Liang, Yane-Shih Wang, Yves S. Y. Hsieh

**Affiliations:** † College of Food Science, Southwest University, Chongqing 400715, PR China; ‡ Division of Glycoscience, Department of Chemistry, School of Engineering Sciences in Chemistry, Biotechnology and Health, Royal Institute of Technology (KTH), AlbaNova University Center, Stockholm SE10691, Sweden; § School of Pharmacy, College of Pharmacy, Taiwan Medical University, Taipei 110, Taiwan; ∥ College of Life Sciences, Shanghai Normal University, Shanghai 220234, PR China; ⊥ Laboratory of Food Chemistry, Wageningen University & Research, Bornse Weilanden 9, 6708 WG Wageningen, The Netherlands; # Division of Organic Chemistry, Department of Chemistry, School of Engineering Sciences in Chemistry, Biotechnology and Health, Royal Institute of Technology (KTH), Stockholm SE1004, Sweden; ¶ Institute of Biotechnology and Biochemical Engineering, Graz University of Technology, 8010 Graz, Austria; ∇ College of Pharmacy, National Taiwan University, Taipei 100, Taiwan; ○ Institute of Biological Chemistry, Academia Sinica, Taipei 11529, Taiwan

**Keywords:** cellulose, lytic polysaccharide monooxygenase, iron oxide, photocatalysis, degradation

## Abstract

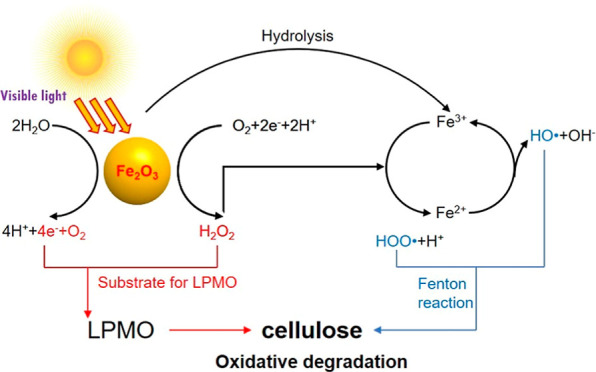

To transform cellulose from biomass into fermentable sugars for biofuel production requires efficient enzymatic degradation of cellulosic feedstocks. The recently discovered family of oxidative enzymes, lytic polysaccharide monooxygenase (LPMO), has a high potential for industrial biorefinery, but its energy efficiency and scalability still have room for improvement. Hematite (α-Fe_2_O_3_) can act as a photocatalyst by providing electrons to LPMO-catalyzed reactions, is low cost, and is found abundantly on the Earth’s surface. Here, we designed a composite enzymatic photocatalysis–Fenton reaction system based on nano-α-Fe_2_O_3_. The feasibility of using α-Fe_2_O_3_ nanoparticles as a composite catalyst to facilitate LPMO-catalyzed cellulose oxidative degradation in water was tested. Furthermore, a light-induced Fenton reaction was integrated to increase the liquefaction yield of cellulose. The innovative approach finalized the cellulose degradation process with a total liquefaction yield of 93%. Nevertheless, the complex chemical reactions and products involved in this system require further investigation.

## Introduction

Cellulose is the most abundant biomass on Earth. One of the most important renewable resources for biofuel production is cellulose from the agricultural and forestry sectors.^1^ Transforming cellulosic biomass into fermentable sugars usually involves thermo-chemical pretreatment processes, of which acid, alkali, and steam methods are used to improve the efficiency of enzymatic degradation of cellulosic feedstocks.^2^ Capital investments toward infrastructural and operational costs of the pretreatment plants are the major expenditure of the biorefinery sectors.^3^ In 2010, a new family of the oxidative enzyme, lytic polysaccharide monooxygenase (LPMO), was discovered.^4^ LPMO oxidatively cleaves at the surface of crystalline cellulose, providing auxiliary activity (AA) to assist the glycoside hydrolase (GH)-catalyzed conversion of recalcitrant cellulose into fermentable sugars. This has accelerated the development of commercial LPMO-GH cocktails, minimized thermo-chemical pretreatment processes, and reduced energy consumption and hazardous waste production.

The activity of this metalloenzyme is dependent on its copper-bound “histidine-brace” structure. It was originally thought that its activity is also dependent on the availability of O_2_ as substrates and reducing agents as electron donors. However, multiple studies in recent years have shown LPMO to prefer H_2_O_2_ over O_2_ as a cosubstrate^5^ and that the low catalytic activity observed previously may be due to the lack of endogenous H_2_O_2_.^5,6^ To apply LPMO at an industrial scale, certain limitations must be overcome: (1) not all LPMOs and cellulolytic enzymes work in synergy, some LPMOs do compete with cellulolytic enzymes at the same substrate binding site.^7^ (2) LPMO catalysis requires an external electron donor and molecular hydrogen peroxide to activate its enzyme. In microorganisms, LPMO catalysis may be fueled by external electrons from the cellobiose dehydrogenase.^8^ However, when the catalysis was carried out in vitro, electron donors such as ascorbic acid, gallic acid, or reduced glutathione and molecular oxygen must be supplied continuously to fuel the catalytic reactions.^9^ These practices increased the cost of operation and limited the applicability of LPMO for industrial biorefinery.^10^

Solar energy is an inexhaustible energy source that can harness chemical reactions. In recent years, photocatalysis research and biocatalysis technologies have emerged. In 2016, Cannella et al. demonstrated that chlorophyllin pigment can be used as a light-induced electron donor for LPMO *Tt*AA9E.^11^ An innovative photocatalytic approach was first exploited by Eijsink and co-workers, using a metal oxide photocatalyst (vanadium-doped titanium dioxide, V-TiO_2_) coupled with LPMO.^12^ Recently, a novel inorganic-biological hybrid platform integrating a silicon photocathode and a LPMO have achieved the visible-light-driven oxidation of chitin.^13^

Inspired by its potential photocatalytic capacity, we envisioned that iron(III) oxide α-Fe_2_O_3_ (hematite), which is low cost and abundant on the Earth’s surface, can be further exploited as a cheap and easily accessible photocatalyst to provide electrons through water oxidation to LPMO-catalyzed reactions. However, α-Fe_2_O_3_ exhibits a poor water oxidation ability due to its short hole diffusion length,^14^ short charge carrier lifetime,^15^ low minority charge carrier mobility,^16^ and finite light penetration depth.^17^ Nevertheless, synthetic nanostructured Fe_2_O_3_ can mitigate these problems by improving the charge transport to the surface in the smaller particles; also, chemically synthesized particles have a significantly lower electrochemical overpotential for water oxidation than bulk particles.^18^ In addition, the α-Fe_2_O_3_ nanoparticle is stable and has d–d electron transition at the wavelengths in a visible-light band gap at 2.06 eV (600 nm), with a direct band gap of 3.3 eV (375 nm).^18,19^ A recent study has shown that cobalt-doped α-Fe_2_O_3_ nanoparticles were successfully used as a photoelectrode material in photoelectrochemical water oxidation.^20^

**Scheme 1:**
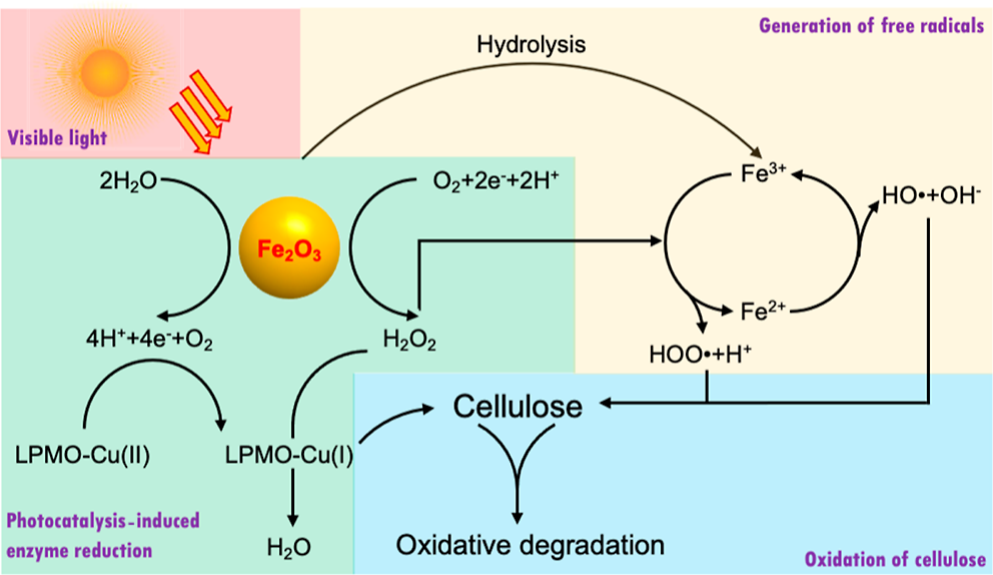
Nano-α-Fe_2_O_3_-Induced LPMO Photocatalysis and Fenton Reaction for Cellulose Degradation

We hypothesized that α-Fe_2_O_3_ nanoparticle-mediated photocatalytic water oxidation can act as an electron donor system by supplying electrons to the LPMO catalytic reaction. Nano-α-Fe_2_O_3_ has a great potential to replace expensive metal oxides or biological chlorophyll in the photocatalytic water oxidation process. Thus far, there is no study integrating α-Fe_2_O_3_ and LPMO biocatalytic reaction. To test our hypothesis, we designed a composite enzymatic photocatalysis–Fenton reaction system using nano-α-Fe_2_O_3_. We evaluated the feasibility of using nano-α-Fe_2_O_3_ as a composite catalyst to facilitate LPMO-catalyzed cellulose oxidative degradation in water. In the α-Fe_2_O_3_ nanoparticle–LPMO system, photocatalytic water oxidation replaces the small-molecule reducing agent (such as ascorbic acid) to generate reducing equivalents (electron–hole pairs are formed on the surface of α-Fe_2_O_3_ nanoparticles), which triggers a series of reactions, including the reduction of LPMO–Cu(II) to LPMO–Cu(I),^12^ O_2_ to H_2_O_2_, and H_2_O_2_ to H_2_O^21^ (Scheme 1). In addition, we integrated a subsequential light-induced Fenton reaction using α-Fe_2_O_3_ as substrate under acidic conditions to generate oxidative hydroxyl radicals and hydroperoxyl radicals. We anticipated that these radicals can induce secondary oxidative degradation of cellulosic materials.

## Materials and Methods

### Preparation of α-Fe_2_O_3_ Nanoparticles

For the synthesis of α-Fe_2_O_3_ nanoparticles, 50 mmol FeCl_3_·6H_2_O (Sigma-Aldrich) was dissolved in 250 mL of 2 mM HCl at 100 °C with stirring. After boiling for 30 min, the solution was cooled to room temperature and α-Fe_2_O_3_ nanoparticles were formed during this procedure.^18^ The remaining ions in the solution were removed by washes with distilled water and centrifugation.

### Expression and Purification of Recombinant *Cm*AA10

Recombinant *Cm*AA10 from *Cellvibrio mixtus* (NCBI reference sequence: WP_039915213.1) was produced and purified according to the protocols previously described.^22^ In brief, the recombinant *Escherichia coli* BL21 star (DE3) cells were grown in Luria Bertani (LB) broth + kanamycin (50 mg/L) at 37 °C on an orbital shaker (200 rpm) until the bacterial density reached OD600 = 0.6–0.8. Protein production was induced by 0.5 mM isopropyl β-d-1-thiogalactopyranoside (Amresco, OH, USA) at 16 °C and 180 rpm for 18 h; the cells were harvested by centrifugation (4000*g*, 15 min). The recombinant protein was released by “osmotic shock”.^23^ Cell pellets were resuspended in a 30 mM Tris-HCl (pH 8) buffer containing 1 mM ethylenediaminetetraacetic acid and 20% (w/v) sucrose at a ratio of 1:50 (wet cell weight/volume in mL). Then, the mixture was agitated at room temperature for 10 min and the cells were recovered by centrifugation (16,000*g*, 30 min at 4 °C). Cell pellets were rapidly resuspended in ice-cold water, agitated for 10 min, and centrifuged (16,000*g*, 30 min). The supernatant containing periplasmic proteins was harvested and passed through an affinity HisTrap column (GE Healthcare). The target protein was eluted with a gradient of increasing imidazole concentration. The purified recombinant protein was concentrated using an Amicon ultracentrifugal filter unit (molecular weight cutoff value of 10,000 Da, Millipore), and the protein concentration was determined using the Bradford assay (Bio-Rad, CA, USA). Purified LPMO was saturated with copper by incubation with a threefold molar excess of CuCl_2_ for 1 h at 30 °C before use. Excess salt was removed by using a PD MidiTrap G-25 desalting column (GE Healthcare).

### Fe_2_O_3_ Nanoparticle-Induced Photocatalytic Reaction

A transparent glass tube (2 mL) was used as a photocatalytic reactor with a reaction volume of 1 mL. The enzyme concentration and substrate (i.e., phosphoric acid swollen cellulose, PASC; prepared as described by Zhang et al.^24^) concentration were fixed at 1 μM and 2% (w/v), respectively. Different amounts of Fe_2_O_3_ were added to 50 mM sodium phosphate buffer (pH 6.0), and the group with 1 mM ascorbic acid was used as a positive control. A 300 W Xe short arc lamp (PerkinElmer model PE300UV) was used to simulate a solar light source. Light-emitting diode (LED) light irradiation was operated under a light source of white LED light (λ > 400 nm, color temperature 6000–6500 K). The vials were laid down on a thermal mixer, and the reaction conditions were set at 200 rpm at 30 °C. The distance between the light source and the sample was approximately 10–15 cm, based on 100 mW cm^–2^ measured at the intensity perceived by the mixture using a light-intensity meter.

### MALDI-TOF-MS Analysis

Qualitative analyses of the enzymatic reaction products were performed by MALDI-TOF MS (Applied Biosystems, CA, USA) according to our previous method.^25^ The reaction product (5 μL) was mixed with 10 mM NaCl (3 μL) and 2,5-dihydroxybenzoic acid (10 mg/mL, 5 μL) in 50% (v/v) acetonitrile.^26^ Then, 1 μL of the mixture was spotted onto a stainless-steel plate and rapidly dried under vacuum for homogeneous crystallization. The spectrometry was performed using an accelerating voltage of 20,000 V with a delay time of 200 ns. The spectrometer was operated in the linear mode.

### HPAEC-PAD Analysis of the Reaction Products

The aldonic products were analyzed using high-performance anion exchange chromatography (HPAEC) using an ICS-5000 system (Dionex, Sunnyvale, CA, USA) equipped with a CarboPac PA-1 column (2 mm ID × 250 mm; Dionex) in combination with a CarboPac PA guard column (2 mm ID × 50 mm; Dionex).^4,27^ The system was further equipped with pulsed amperometric detection (PAD). Two mobile phases (A) 0.1 M NaOH and (B) 1 M NaOAc in 0.1 M NaOH were kept under helium flushing and a column temperature of 20 °C. The elution profile applied was as previously described.^28^

### Quantitative Analysis of the Introduced Carboxylate Functionality

Based on our previous report,^22^ carboxymethylcellulose (CM-cellulose) was added into 540 μL of ethanol/4-(2-hydroxyethyl)-1-piperazineethanesulfonic acid (HEPES) buffer (ethanol/10 mM HEPES, pH 8 = 95:5, v/v), giving CM-cellulose concentrations from 1 to 7 mM. The solutions were mixed well and left undisturbed for 2 min. NiCl_2_ in ethanol/HEPES buffer (2 mM, 60 μL) was then added to the CM-cellulose solutions. After vigorous mixing, the solutions were left at ambient temperature for 2 min and then centrifuged (16,000*g*, 5 min) to precipitate Ni^2+^–CM-cellulose particles. The supernatant (500 μL) was removed and mixed vigorously with pyrocatechol violet (PV) (500 μL, 80 μM), giving a final PV concentration of 40 μM. The absorbance was recorded immediately. A standard curve of CM-cellulose was plotted with a known degree of substitution using absorbance and concentration of carboxyl group (in the initial 600 μL = 540 μL of buffer + 60 μL of 2 mM NiCl_2_) derived from the equation.^22^ The relative amounts of carboxyl groups on LPMO-treated PASC were quantified and compared with the CM-cellulose standard curve.

### Fenton Reaction

Following the photocatalytic reaction, an optimal concentration of 240 mM HCl was added to the reaction system which contains 5 mg/mL Fe_2_O_3_ nanoparticles and the mixture was agitated at 30 °C for 24 h until all the iron oxide was converted into ferric ions (confirmed by no visible reddish-brown precipitation after centrifuge). The mixture was adjusted to pH = 2 with sodium hydroxide. Hydrogen peroxide was then added to the reaction system at a concentration of 25 times that of the ferric ions.^29^ The Fenton reaction was carried out under the same photocatalysis light source for 24 h. The supernatants were analyzed by MALDI-TOF-MS. The insoluble fraction was obtained by centrifugation and removal of the supernatant. The liquefaction yield was expressed as
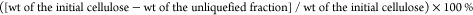


## Results and Discussion

### Evaluation of α-Fe_2_O_3_-*Cm*AA10 Photobiocatalysis

**Figure 1:**
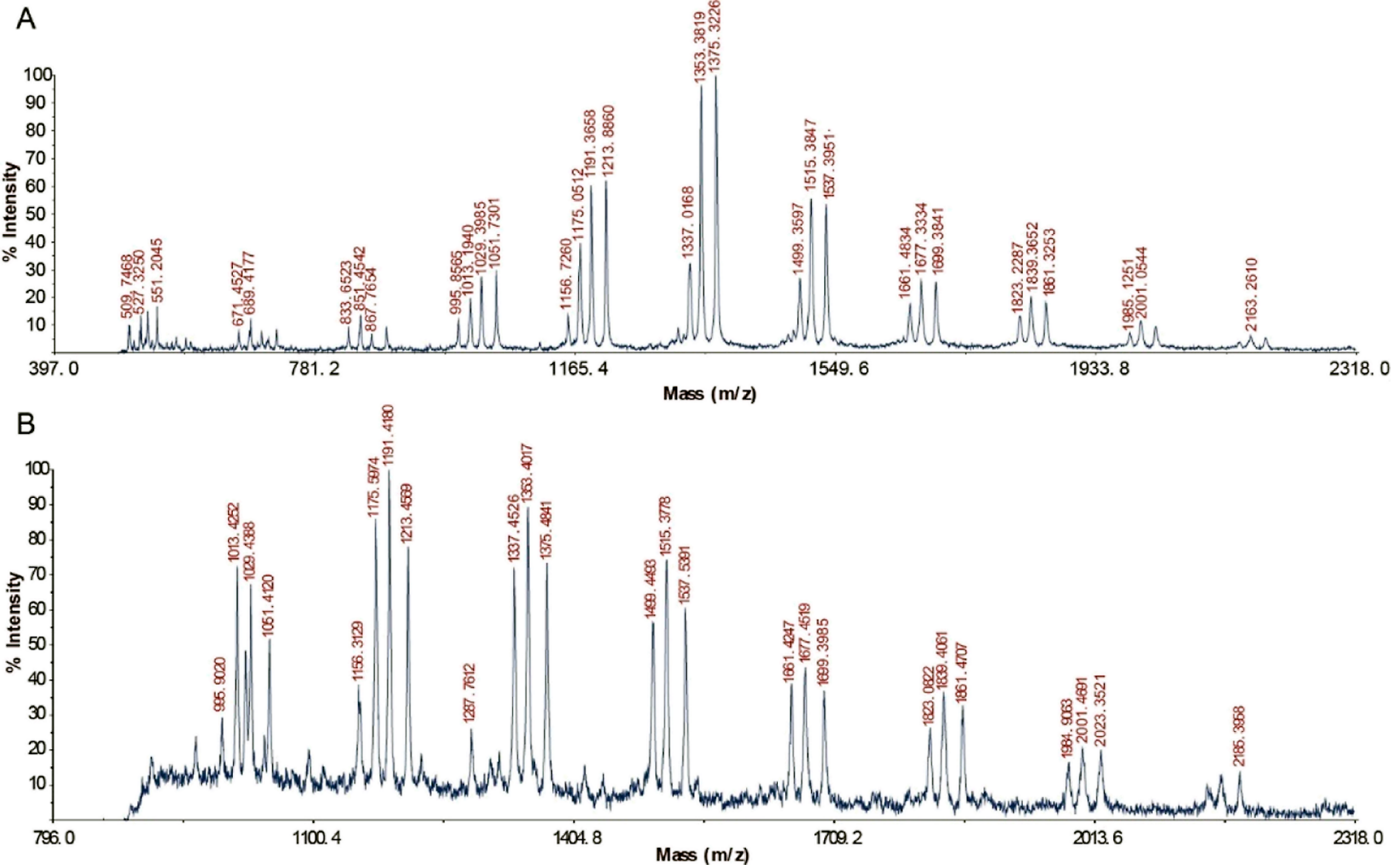
MALDI-TOF-MS analysis of the reaction products of *Cm*AA10 and PASC with (A) ascorbic acid and (B) α-Fe_2_O_3_ under visible light as electron donors. Ions with *m*/*z* of 867, 1029, 1191, 1353, 1515, 1677, 1839, 2001, and 2163 represent oligosaccharides DP5-DP13 in a lactone form; 889, 1051, 1213, 1375, 1537, 1699, 1861, and 2185 represent oligosaccharides DP5–DP13 in an aldonic acid form; 527, 689, 851, 1013, 1175, 1337, 1499, 1661, 1823, and 1985 represent cello-oligosaccharides DP3–DP12; 509, 671, 833, 995, and 1157 represent dehydrated oligosaccharides DP3–7 formed by phosphoric acid treatment.

In this study, we tested whether α-Fe_2_O_3_ nanoparticles can be used as a photocatalyst to provide electrons for the oxidation reaction of LPMO. First, we tested the α-Fe_2_O_3_-*Cm*AA10 reaction system under visible light by comparing it with the control group where electrons were supplied by ascorbic acid. MALDI-TOF MS analysis revealed *Cm*AA10 to degrade PASC oxidatively into cello-oligomers with a degree of polymerization from DP3 to DP9 in the presence of ascorbic acid (Figure 1A). For *Cm*AA10 supplied with α-Fe_2_O_3_ nanoparticles (Figure 1B), we found similar oxidized product patterns, confirming that α-Fe_2_O_3_ nanoparticles can serve as electron donors for the LPMO catalytic reaction.

**Figure 2:**
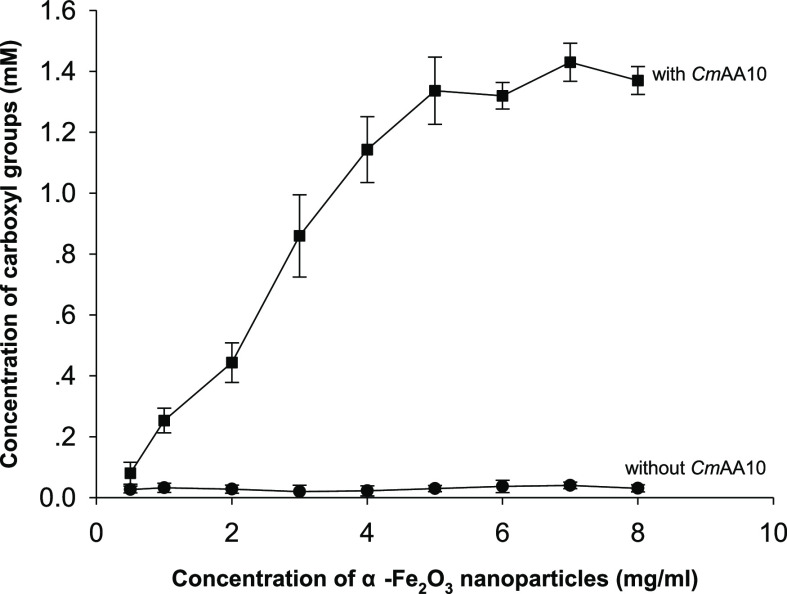
Carboxyl group formation under different concentrations of α-Fe_2_O_3_ in the LPMO photocatalysis.

**Figure 3:**
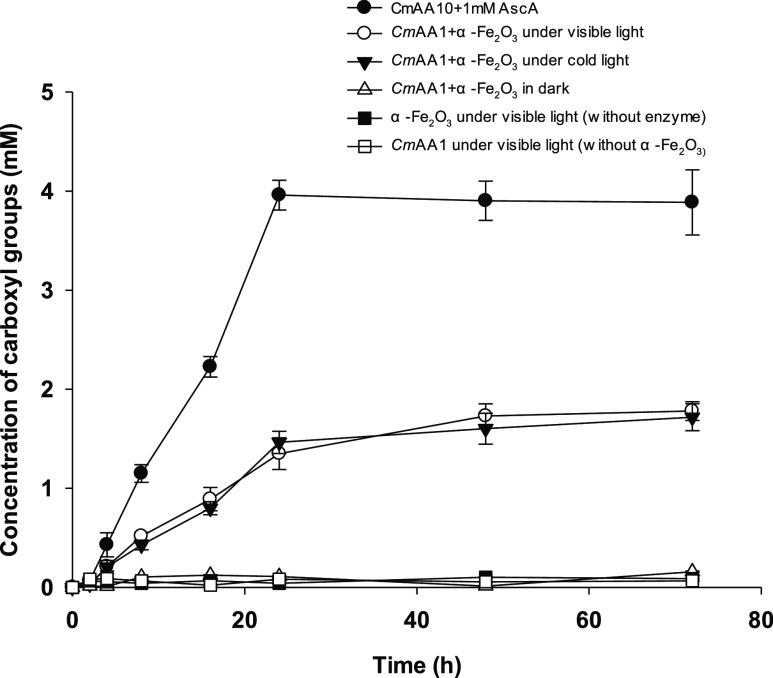
Carboxyl groups formation of the α-Fe_2_O_3_-LPMO photocatalysis system under different conditions. Generation of ferric ions for the subsequent Fenton reaction.

We further optimized the α-Fe_2_O_3_ concentration used in this photocatalysis system by relative comparison of the carboxyl groups introduced by the LPMO on the insoluble cellulose. We found the iron oxide concentration from 0.5 to 5 mg/mL to be positively correlated with the reaction rate when 2% PASC was used as a substrate, and the photocatalysis reaction reached saturation at approximately 5 mg/mL nano-α-Fe_2_O_3_ (Figure 2). We were interested in how the system would behave in a real-life environment, such as under sunlight, so we simulated the reaction by exposing the photocatalytic system under normal sunlight intensity (100 mW cm^–2^), using a 300 W Xe short arc lamp over 80 h exposure. The reaction reached the maximum yield of 1.3 mM carboxylate moiety introduced within 24 h, which is 3-fold less than the value obtained in the control reaction (i.e., 4 mM using ascorbic acid as the electron donor). We speculated that nano-α-Fe_2_O_3_ had triggered photocatalytic water oxidation, and as a result, oxygen and hydrogen peroxide were generated,^30^ which in turn became substrates to the enzymatic reaction and lowered the reaction yield. It has been reported that the over-production of reactive oxygen species (ROS) such as H_2_O_2_, superoxide, and hydroxyl radicals generated via oxygen reduction could negatively affect LPMO stability.^9,31^ Although these ROS are ideal substances desirable for the second reaction step in our design, it is necessary to limit the light intensity to keep water oxidation within a “safe” range so that the enzymatic reaction can proceed. We found it interesting that the LED cold light lamps which did not contain infrared radiation could also lead to a catalytic effect similar to that of visible light (Figure 3). Therefore, for the conditions that require the use of artificial light sources, the use of LEDs over arc lamps offers several advantages, such as less energy consumption, 10–100 times lower in price compared to arc lamps, and a much longer lifespan (50,000 h vs 1000 h).^32^

**Figure 4:**
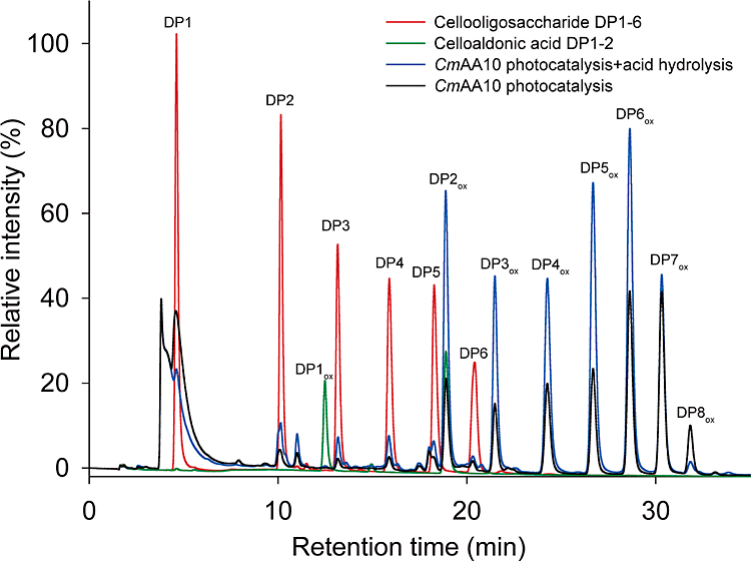
HPAEC-PAD oligosaccharide profiles before and after acid treatment of α-Fe_2_O_3_.

Some fungal species are able to degrade lignocellulosic biomass using Fenton chemistry.^33^ The Fenton reaction involves the oxidation of Fe^2+^ to Fe^3+^ by H_2_O_2_ (Scheme 1), forming a hydroxyl radical (HO^•^) and a hydroxide ion (OH^–^), and the reduction of Fe^3+^ to Fe^2+^ by H_2_O_2_ to form a hydroperoxyl radical (HOO^•^) and a proton (H^+^). H_2_O and O_2_ are also generated during the process. These oxygen-free radicals can lead to the oxidative degradation of lignocellulosic substances and generate other ROS in the process.^31,34^ In our system, the photocatalyst α-Fe_2_O_3_ was subsequently converted into Fe^3+^ ions by hydrochloric acid. By minimizing the HCl loading amount, 240 mM HCl was applied for the total conversion of Fe_2_O_3_ to FeCl_3_. Under optimized conditions, acid hydrolysis of cellulose also occurred (Figure 4). HPAEC-PAD analysis revealed that glucose, gluco-oligosaccharides DP2 to DP6, and aldonic acids DP2 to DP8 were produced by *Cm*AA10 photocatalysis. Furthermore, the relative proportions of cellulose oligosaccharides and aldonic acids significantly increased after the acid treatment. This shows that while HCl is used to decompose Fe_2_O_3_ to obtain Fe^3+^ for the subsequent Fenton reaction, the soluble cello-oligosaccharides, aldonic acids, and insoluble cellulose residues may also be hydrolyzed by acid, resulting in the increase of both lower DP oligosaccharides and aldonic acids.

### Impact of the Fenton Reaction after LPMO Photocatalysis on Cellulose Degradation

The Fenton reaction is widely used in the degradation of toxic substances in sewage treatment and other fields. It has the advantage of not relying on high temperature, high pressure, and high concentration of chemical substances. Recently, it has been applied to biomass pretreatment, especially lignocellulose. The Fenton reaction has been proven to improve the subsequent enzymatic digestibility of cellulose and hemicellulose.^29,35,36^

**Figure 5:**
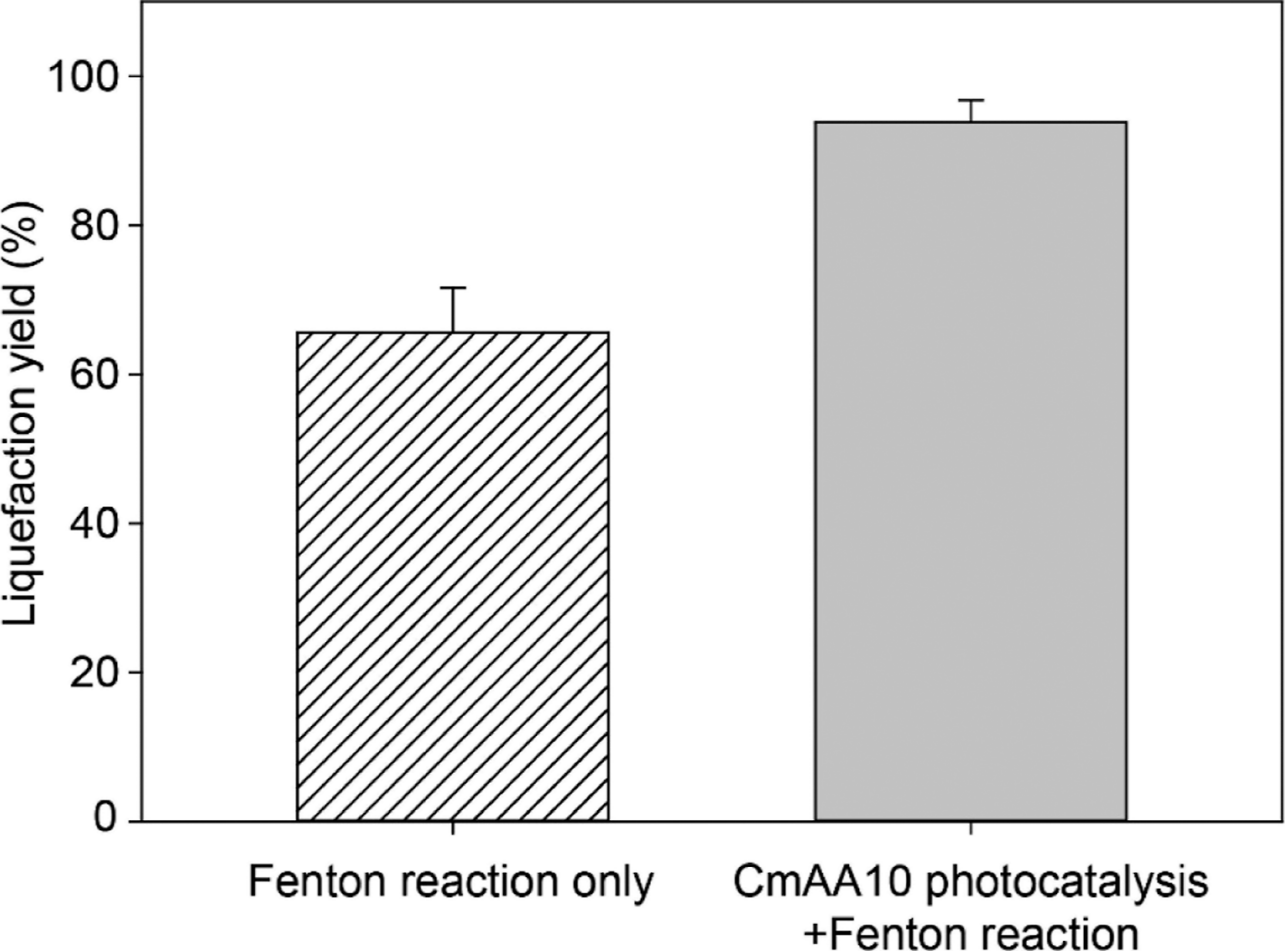
Liquefaction yields calculated based on the remaining weight of insoluble cellulose.

After the acid treatment of α-Fe_2_O_3_ with HCl, the rust-colored Fe_2_O_3_–cellulose mixture became a pale-yellow FeCl_3_–cellulose suspension. The pH of the reaction system was adjusted to pH 2 to avoid the consumption of hydrogen peroxide and the formation of iron hydroxide. For H_2_O_2_ used in the Fenton reaction, an optimal Fe^3+^–H_2_O_2_ ratio of 1 to 50 was applied.^29^ After 24 h of reaction, the transparency of the originally opaque cellulose suspension increased significantly (Figure S1A,B), proving that a large amount of insoluble cellulose was converted into soluble oxidized oligosaccharides and other small molecular compounds in the oxidation reaction initiated by Fenton’s reagent. We further quantified the liquefaction yield of the system. Although the Fenton reaction alone under the same experimental conditions (i.e., Fe^3+^ concentration, hydrogen peroxide concentration, and pH) reached a liquefaction yield of 65% (Figure 5), the results showed the yield obtained after the Fe_2_O_3_-LPMO treatment was as high as 93%. It is obvious that after LPMO photocatalysis, cellulose had become easier to be oxidized and degraded by Fenton’s reagent, with the liquefaction yield increased by 28%, we speculate that this is because the oxidation reaction of LPMO and the ROS generated by photocatalytic water oxidation destroyed the crystal structure of the cellulose. Similar to the fact that LPMO can promote the efficiency of subsequent hydrolase reactions, the introduction of “scratches” and carboxyl groups on the crystal surface leads to the formation of loose structures, providing more opportunities for subsequent reactions. The MALDI-TOF-MS snapshot indicates that the oligosaccharide fragments formed during this reaction mainly come from oxidative degradation (Figure S1C), with a 176 Da molecular weight interval between oligosaccharides with different degrees of polymerization, suggesting that the hydroxyl group of each glucose building block was transformed to a carboxyl group by the oxidative reaction. The mass spectrometry profile also indicates the presence of other molecules, and further investigations are necessary to identify the oxidative degradation products obtained from the Fenton reaction.

So far, we have explored the role of α-Fe_2_O_3_ as a photocatalyst to replace the small-molecule reducing agent as the electron donor of LPMO in the degradation of cellulose. Compared with other metal oxides or biological photocatalysts, the catalytic efficiency of iron oxide as an electron donor for LPMO may not be the most prominent, but as a functional photocatalyst, its low cost and easy availability allow application upscaling. More importantly, we also explored the possibility of decomposing α-Fe_2_O_3_ into ferric ions for use in the subsequent Fenton reaction. In the previous studies, the Fenton reaction was often applied to biomass as pretreatment before the enzymatic reaction to improve enzymatic hydrolysis efficiency. In this study, we have demonstrated that the oxidative degradation by LPMO photocatalysis followed by the Fenton reaction can even increase the liquefaction yield of cellulose.

## Conclusions

Metal oxide-based photocatalysis is an innovative approach to substitute the use of small-molecule reductants for providing external electrons required in LPMO catalysis. Our study confirmed the starting hypothesis that α-Fe_2_O_3_ nanoparticles can act as an electron donor to provide electrons to the LPMO catalytic reaction. Here, we report a novel integrated process for the degradation of cellulose by combining LPMO-catalyzed oxidative degradation with the Fenton reaction. Hematite (α-Fe_2_O_3_)-mediated photocatalysis displays a reductant function for LPMO *Cm*AA10, resulting in an effective oxidative degradation of PASC with a product profile similar to that obtained with ascorbic acid. The subsequent Fenton reaction finalizes the degradation process to obtain a total liquefaction yield of 93%. Our research has undoubtedly enriched the selection range of photocatalysts applicable in LPMO-catalyzed reactions. The introduction of the Fenton reaction has further amplified the role of hematite while enhancing its ease of use. Related complex chemical mechanisms and various products in this system are currently under investigation.

## Supporting Information Available

Effect of LPMO photocatalysis–Fenton reaction on cellulose liquefaction (PDF)
