# Supplementary material for: Novel Two-Step Process in Cellulose Depolymerization: Hematite-Mediated Photocatalysis by Lytic Polysaccharide Monooxygenase and Fenton Reaction
Source: J Agric Food Chem. 2022 Aug 3;70(32):9941–7. doi: 10.1021/acs.jafc.2c02445 (PMC9389612; doi:10.1021/acs.jafc.2c02445)
Supplement: Supplementary file 1 — jf2c02445_si_001.pdf [file jf2c02445_si_001.pdf]

Supporting Information

A novel two steps process in cellulose depolymerization: hematite-mediated photocatalysis by lytic polysaccharide monooxygenase and Fenton reaction

Damao Wang*[a,b,c], Mu-Rong Kao[c], Jing Li[b,d], Peicheng Sun[e], Qijun Meng[f], Anisha Vyas[b,g], Pi-Hui Liang[h], Yane-Shih Wang[i], Yves S. Y. Hsieh*[b,c]

[a] College of Food Science, Southwest University, Chongqing, 400715, PR China

[b] Division of Glycoscience, Department of Chemistry, School of Engineering Sciences in Chemistry, Biotechnology and Health, Royal Institute of Technology (KTH), AlbaNova University Center, Stockholm, SE10691, Sweden

[c] School of Pharmacy, College of Pharmacy, Taipei Medical University, Taipei 110, Taiwan

[d] College of Life Sciences, Shanghai Normal University, Shanghai 220234, PR China

[e] Laboratory of Food Chemistry, Wageningen University & Research, Bornse Weilanden 9, 6708 WG Wageningen, The Netherlands

[f] Division of Organic Chemistry, Department of Chemistry, School of Engineering Sciences in Chemistry, Biotechnology and Health, Royal Institute of Technology (KTH), Stockholm, SE1004, Sweden

[g] Institute of Biotechnology and Biochemical Engineering, Graz University of Technology, 8010, Graz, Austria

[h] College of Pharmacy, National Taiwan University, Taipei 100, Taiwan

[i] Institute of Biological Chemistry, Academia Sinica, Taipei 11529, Taiwan

* Corresponding author: Yves S. Y. Hsieh; E-mail: yvhsieh@kth.se

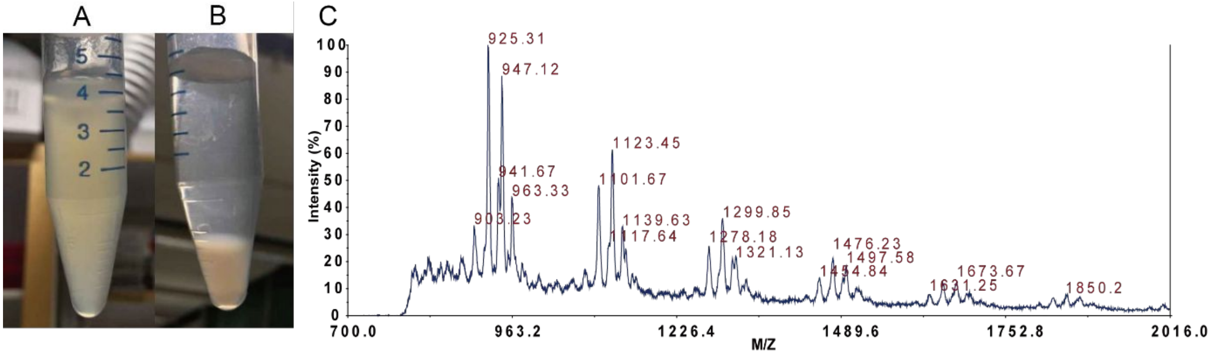

Fig. S1 Effect of LPMO photocatalysis-Fenton reaction on cellulose liquefaction. After LPMO photocatalysis, the solution was treated A) without or B) with Fenton reaction, C) MALDI-TOF-MS snapshot of cellulose degradation products using LPMO photocatalysis-Fenton reaction.
